# A participatory qualitative study exploring prioritization of climate change and health research among women in Indonesia and Malaysia

**DOI:** 10.3389/fpubh.2026.1789137

**Published:** 2026-07-20

**Authors:** Raksha Pandya-Wood, Dessy Rosalina, Gabriela Fernando, Grace Wangge, Savyna Selvarajan, Azliyana Azhari, Nadira R. Chairani

**Affiliations:** 1 Translational Health Research Institute, Western Sydney University, Sydney, NSW, Australia; 2 Monash Climate Communication Hub, Monash University, Bandar Sunway, Malaysia; 3 Monash Climate Communication Hub, Monash University, Bumi Serpong Damai, Indonesia; 4 Public Health, Monash University, Bumi Serpong Damai, Indonesia

**Keywords:** climate change, global south, health, research priorities and challenges, women

## Abstract

In Indonesia and Malaysia, women face disproportionate climate-related health impacts due to intersecting social, economic and environmental inequalities. Climate and health policies overlook these gendered vulnerabilities, highlighting the need for approaches that center women’s lived experiences and priorities. This qualitative study identifies and ranks women’s priority climate-related health risks. We conducted participatory focus group discussions with 35 women recruited via snowball and purposive sampling in Indonesia (*n* = 21) and Malaysia (*n* = 14), incorporating a structured ranking exercise informed by the Centres for Disease Control climate–health framework. A hand-coded thematic analysis approach was used. For Indonesian women, air pollution (e.g., respiratory issues) ranked highest, while for Malaysian women, extreme heat (e.g., leading to dizziness) ranked highest. Participants explained their reasons for contextualizing their rankings, such as living in urban or rural areas in Indonesia and Malaysia. These findings provide critical, nuanced insights into the urgent need to better consider gendered dimensions and to develop more gender-responsive climate policies that center the lived experiences of women regarding the climate-health nexus in Southeast Asia. As climate impacts intensify, ensuring that women’s voices inform both national and international policy frameworks is not only a matter of equity but a prerequisite for effective and sustainable public health responses.

## Introduction

Climate change refers to long-term shifts in global temperatures and extreme weather patterns driven by fossil fuel combustion, deforestation, and industrial emissions (1). These shifts contribute to broader environmental degradation, encompassing the deterioration of ecosystems, loss of biodiversity, and depletion of natural resources (2). Southeast Asia is among the world’s most climate-vulnerable regions, facing rising sea levels, intensifying typhoons, and prolonged droughts that threaten food, water, and livelihood security. Vulnerabilities are deepened by the region’s high population density, dependence on climate-sensitive sectors, and limited adaptive capacity (3). While these concepts are closely interconnected, we distinguish between climate change, environmental degradation, and environmental health impacts to enhance conceptual clarity. Climate change refers to large-scale, long-term shifts in climate systems driven primarily by anthropogenic greenhouse gas emissions. Environmental degradation is understood as the deterioration of ecosystems and natural resources such as deforestation, pollution, and biodiversity loss which may be both a consequence of and contributor to climate change. Environmental health impacts refer to the effects of these processes on human health, including outcomes such as air pollution-related respiratory illness, heat stress, and waterborne disease. In this study, we examine how women experience these interconnected processes in their daily lives, recognizing that they might perceive and articulate these as overlapping rather than discrete categories.

### Indonesia and Malaysia’s vulnerability to climate change

Indonesia’s geography and ecosystems make it highly vulnerable to climate change. With thousands of islands spanning the Pacific and Indian oceans, its coastal and inland communities face major health risks from environmental degradation (4). Air pollution, sea level rise, heat, and extreme weather events, such as floods and droughts, disproportionately harm rural and low-income populations especially women dependent on fragile resources (5). Indigenous and coastal groups are at increased risk of economic, health, and cultural impacts due to climate-sensitive resources and disaster-prone locations. Despite their heightened exposure, women are often excluded from decision-making in climate adaptation, emphasizing the need for gender-transformative resilience (6–8).

Similarly, neighboring Malaysia faces climate-induced hazards such as floods and heatwaves, which pose significant threats to public health, especially among women living in poverty with limited access to healthcare and adaptive infrastructure. Among vulnerable groups, rural women are exposed to unsafe water sources, and Indigenous populations face vector-borne diseases in remote areas (9).

These gender and health concerns are echoed in recent research. Awareness of climate change among women in Indonesia (10) and Malaysia is high (11), yet gender-specific integration into climate policy remains limited. Indonesian women report significant concern and willingness to act, though specific segmentation data is lacking. In Malaysia, a national survey found deep climate concern among women, yet national climate strategies often omit a gendered health perspective, hindering targeted, effective action (39). Global policy frameworks, such as the UNFCCC Gender Action Plan, emphasize the importance of gender-responsive climate action and the meaningful participation of women in climate decision-making processes (12).

With a regional view, the demographic landscape of Southeast Asia’s 350 million women, particularly in Malaysia and Indonesia, raises critical questions about how climate change uniquely affects women and girls across the life course (Table 1).

**Table 1:** Demographic breakdown of the female population in Indonesia and Malaysia.

**Age group**	**Malaysia (millions)**	**Indonesia (millions)**
Girls under 12	3.11	31.1
Women 13–25	4.85	28.4
Women 26–45	4.34	41.2
Women 46–65	3.41	29.8
Women 65 and above	1.37	13.1

For girls under age 12 in both countries, climate change challenges include ensuring safe transportation to school during haze periods, floods, and extreme heat (13), as well as ensuring girls acquire survival skills such as swimming (14). Additionally, school uniform policies may need to consider cultural practices, such as wearing the *tudung* (headscarf), and use more breathable fabrics to prevent overheating (15). Adolescent and young women aged 13–25 years (approximately 28.4 million women) experience monthly menstrual cycles and may face menstrual inequity caused by climate change, which can hinder access to clean water, sanitation, and menstrual hygiene (16). Access to reproductive health services might be limited during floods or landslides, blocking access to essential clinics (17). Some women also juggle work, household duties, and caring for their older parents and children (18). Women aged 46–65 are mostly undergoing perimenopause and postmenopause, phases marked by hormonal fluctuations and physiological changes that can affect their ability to regulate body temperature. Overall, across the female life course, the interaction among biological changes, social norms and expectations, and the structural barriers women face in the context of climate change remains poorly understood. Furthermore, in both Indonesia and Malaysia, women’s primary role in caregiving increases their exposure to climate hazards, as they are more likely to remain in high-risk areas during extreme weather events while caring for children, older adults, and unwell family members.

### Health risks in Indonesia and Malaysia

Southeast Asian literature highlights that climate change puts women at higher risk, particularly for respiratory and heat-related illnesses. In urban areas like Jakarta, Indonesia, more than 10.5 million people face significant threats from air pollution, especially due to the country’s highest annual ambient PM2.5 concentrations. Syuhada et al., (19) estimate air pollution causes over 7,000 adverse health effects in children, more than 10,000 deaths, and over 5,000 hospitalizations each year. These health impacts add to women’s burdens, as they often care for their families. Women are more likely to work outdoors in agricultural and informal sectors, exposing them to heatwaves (9). They also experience higher psychological impacts from climate-related disasters, such as post-traumatic stress disorder (PTSD). Disasters such as droughts, aridity, and floods have been linked to increased child marriage and adolescent births [(20), p. 3]. Additionally, 28% of women face barriers to healthcare access, mostly due to cost and safety concerns (20). Marginalized groups including the urban poor, single mothers, the disabled, and older women face greater vulnerabilities because social safety nets are often inadequate and they live in informal settlements exposed to climate hazards. Indigenous peoples also face these challenges. *Orang Asli* are Indigenous minorities in Peninsular Malaysia, and *Orang Asal* includes all Indigenous peoples in Malaysia, including those in Sabah and Sarawak (East Malaysia). In Indonesia, *Masyarakat Adat* are particularly vulnerable to vector-borne diseases like malaria, as well as zoonotic diseases (21). Remote coastal and mountainous regions create challenges for public health surveillance (20). Rural women often lack access to clean water and must use harmful cooking fuels, raising the risk of malnutrition, cholera, and respiratory illnesses (20). For rural Indigenous women, these health risks are linked to daily socio-economic conditions: limited or insecure land rights reduce their ability to adapt farming and food practices to new rainfall patterns; reliance on forest and coastal resources makes them highly vulnerable to ecosystem damage; and linguistic and geographic barriers to formal health services cause many climate-sensitive illnesses to go undetected and untreated. Urban-focused climate-health indicators often fail to capture these issues. Age and gender continue to influence health outcomes. Deaths from heat-related events, such as cardiovascular, cerebrovascular, and respiratory conditions, affect older people more. Transgender individuals face discrimination in accessing healthcare, which increases their vulnerability to sexually transmitted infections and other health risks in a changing climate (22).

### Lack of disaggregated data

The lack of disaggregated data and the marginalization of women’s voices in decision-making have further obstructed the development of inclusive climate-health solutions. Neglecting women’s specific health burdens, such as reproductive and mental health issues worsened by disaster-related displacement or loss, raises major ethical concerns (23). These patterns underscore the urgent need for intersectional, gender-sensitive research. Such research should identify health disparities and inform equitable and context-specific policy responses.

An intersectional approach is crucial for understanding how various axes of identity, such as socioeconomic status, rurality, and caregiving roles, interact and influence women’s exposure to and resilience against climate risks. Drawing on intersectionality theory (24) and feminist climate justice perspectives, this study recognizes that climate-related vulnerabilities are not experienced uniformly but are produced through overlapping systems of inequality. In addition, ecofeminist perspectives highlight the interconnected exploitation of natural environments and women’s bodies, particularly in contexts where livelihoods are closely tied to ecological systems. Together, these frameworks enable a more nuanced understanding of how structural power relations shape climate-health outcomes for women in Southeast Asia. This is especially relevant in Southeast Asia, where patriarchal norms and structural inequalities continue to affect climate adaptation outcomes (25). By prioritizing women’s needs and lived experiences, particularly those from marginalized groups, future research can more effectively guide the co-creation of health interventions and climate policies that are just, inclusive, and sustainable. This study seeks to identify and prioritize research topics at the intersection of climate change and human health, as perceived by women in Malaysia and Indonesia.

### The research gap

Despite growing awareness of climate change’s health impacts, a significant gap persists: participatory research that centers women’s lived experiences in defining climate–health priorities in Southeast Asia remains absent. Most studies address general population risks or policy-level analyses and seldom include women, particularly those who are marginalized or from community-based contexts, as active contributors to setting research priorities. This gap is crucial, as women’s experiences of climate-related health risks are distinct due to intersecting social, economic, and environmental inequalities, which conventional research often misses.

## Methods and conceptual framework

The aim of this work is to inform future research agendas on climate change and health that genuinely reflect the lived experiences and priorities of women. Women from Indonesia and Malaysia are largely excluded from decision-making spaces in the design, conduct, dissemination, and implementation of research. Therefore, we focus solely on their voices here. This study intersects social science and health disciplines. We employ a methodology that combines interpretivism and pragmatism.

### Participant selection criteria

We used a qualitative participatory approach in a focus group setting. This helped shift power dynamics and ensured research was driven by lived experiences rather than external agendas (25). We used purposive, snowball, and non-probability sampling (40). Participants were recruited through NGOs and community organizations involved in climate and health issues.

To ensure participant quality, two main eligibility criteria were established. The primary criteria required participants to be actively employed within community organizations or NGO engaged in issues at the intersection of climate change, women, and health.

The secondary criteria required direct professional involvement in health impact cases attributed to climate change, including: dengue fever or chikungunya (consequent to rising temperatures and increased rainfall intensity); respiratory and cardiovascular diseases (consequent to air pollution and extreme weather events); heatstroke (consequent to extreme heat exposure); and other climate health conditions.

The sample included 14 women in Malaysia and 21 in Indonesia. Participants were selected through purposive and snowball sampling to capture diverse perspectives. We considered age, ability, ethnicity, religion, geographical location (rural and urban), Indigenous backgrounds, employment, marital status, and health backgrounds. Initially, we identified interested participants through our in-country research partners and community networks. Subsequent recruits joined via referrals to reach less accessible individuals. Eligible participants are women aged 18 and above who reside in Malaysia or Indonesia and are willing to participate in group discussions. Efforts ensured balance across these characteristics to reflect varied lived experiences within each country. Men were excluded unless attending as carers. Individuals under 18 or residing outside these countries were also excluded. Transgender communities were invited but were unable to attend because of scheduling conflicts. The study received ethical approval from Monash University (ID: 45509), the National Research and Innovation Agency of Indonesia (Badan Riset dan Inovasi-BRIN, No: 084/KE.01/SK/02/2025), and the World Health Organisation (ID: ERC.0004217). All participants received study information in their preferred language to ensure informed consent.

### Data collection and facilitation

The two full-day workshops in Jakarta and Kuala Lumpur were facilitated by culturally aware facilitators who possessed knowledge of and sensitivity to local cultural norms, languages such as *Bahasa Indonesia*, Tamil, *Bahasa Melayu*, indigenous dialects, and English, as well as traditions and histories, including information on Indigenous communities in Indonesia and Malaysia. The team was supported by note-takers, and audio recordings were made to ensure accurate documentation of discussions. All facilitators and note-takers received prior training in participatory qualitative methods and were briefed on their roles and expectations before each workshop. Facilitators maintained neutrality, encouraged inclusive participation, and clarified concepts using local languages and culturally relevant examples. In total, there were eight facilitators: eight note-takers, all women. The discussions followed a semi-structured format covering: (1) Introduction: purpose, confidentiality, and consent, (2) Warm-up: participants’ experiences with climate change and health in their communities, (3) Exploration: barriers and enablers to involvement in climate and health research, (4) Prioritization: ranking exercise of research topics based on a visual framework, and (5) Reflection: participants’ reasons for their rankings and identification of missing priorities (parts 4 and 5 being the focus of the current article).

Data generated from the workshops in Indonesia were translated verbatim from *Bahasa Indonesia* to English, and data from the Malaysian workshops were translated verbatim from *Bahasa Melayu* to English. Some participants in Malaysia spoke in both English and *Bahasa Melayu*. Van Nes et al., (26) state that when conducting research across multiple languages, interpretation can be fragile because meanings may be interpreted differently. In this context, researchers must identify the most accurate meaning of the statements when analyzing the words and the sentiment behind them to prevent the loss of content during translation (26).

To ensure linguistic rigor and consistency, our team consists of native speakers who facilitated the initial data collection stage (FGDs). Transcription and data analysis were carried out by a research team comprising two Indonesian native speakers and one Malay-speaking researcher. Therefore, our research team analyzed the participants’ language by examining the words used to ensure semantic accuracy. Translation into English was performed after analysis to preserve cultural nuances (27). We were mindful that, when translating data, idiomatic expressions or culturally specific concepts in *Bahasa Indonesia* or Bahasa Malaysia might not have direct equivalents in English. Local metaphors or sayings (e.g., expressions related to weather, health, or social roles) often lose their emotive or social context when translated literally. Similarly, words with multiple layers of meaning (e.g., village, or desa in Indonesia, and kampung in Malaysia) can be oversimplified or inaccurately translated. Additionally, we recognized that English often reinforces Western epistemologies, where knowledge produced in English is regarded as more “legitimate.” Indigenous or locally rooted understandings may be sanitized or depoliticized during translation to align with global policy discourse or academic norms. Consequently, to enrich the socio-cultural depth of participants’ experiences, we retained native quotes alongside their English equivalents during data analysis (28).

### Workshop structure

The workshop included five sequential sessions, each lasting 2 to 3 h. Three sessions validated women’s priorities, and the fourth determined them. This structure helped explore participants’ experiences in depth while ensuring data diversity.

The five sessions are: (1) Introduction to Women’s Identity and Climate, which sets the context for the series; (2) Women’s Roles and Contributions in Climate and Health Research, aiming to highlight women’s involvement in research activities; (3) Overcoming Structural Barriers and Acknowledging Diverse Identities, designed to explore the challenges faced and recognize the spectrum of identities; (4) Research Priority Setting, focused on determining key areas for future investigation; and (5) Recommendations - Translating Priorities into Action, intended to develop actionable next steps from the established priorities.

### Visual framework

**Figure 1:**
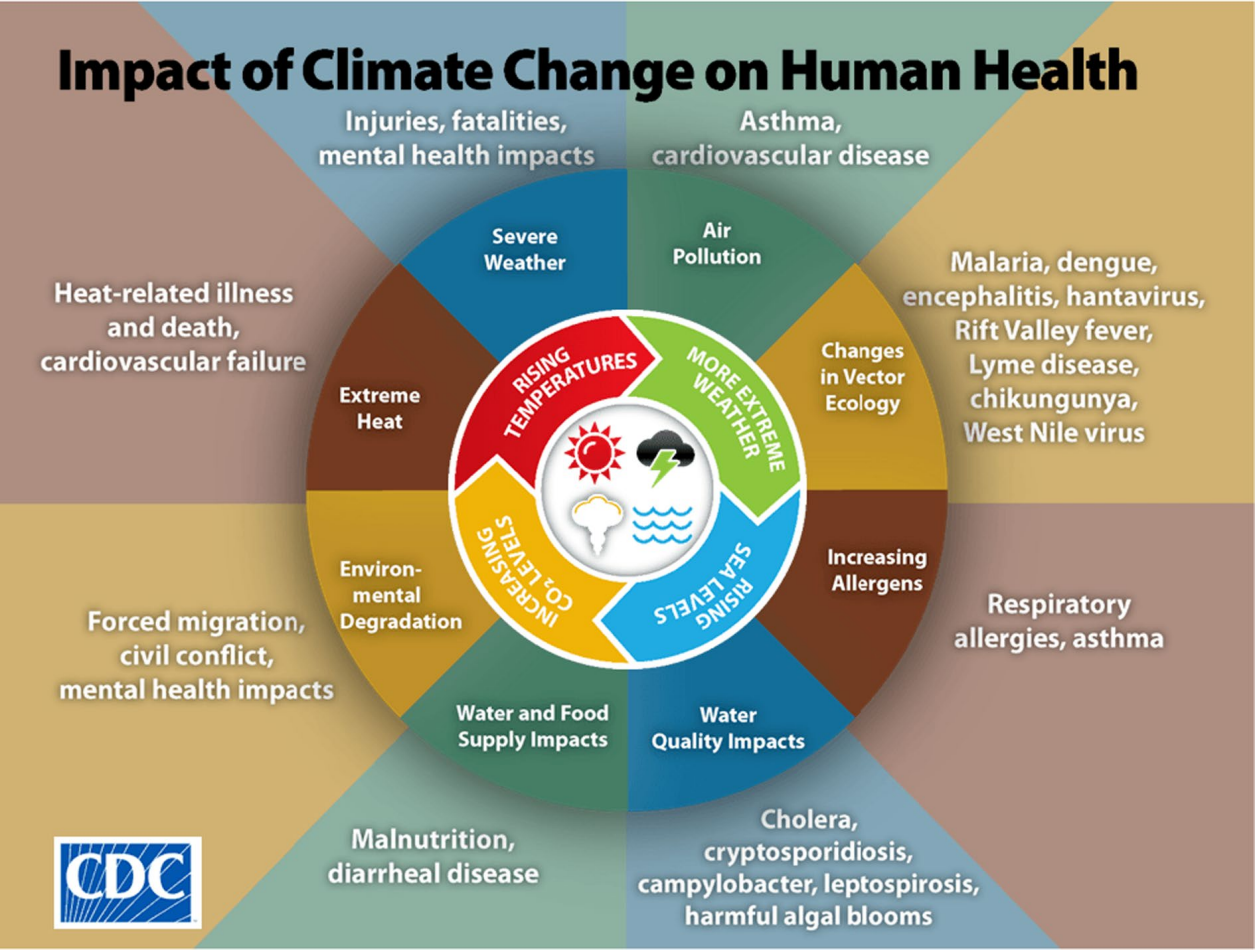
Centers for Disease Control and Prevention (CDC) climate change and health framework (29). Figure: Impacts of Climate Change on Human Health. Source: Centers for Disease Control and Prevention (CDC), U.S. Department of Health and Human Services. This image is a work of the United States Federal Government and resides in the public domain. Image unmodified from the original.

To guide discussions and prioritization, we verbally translated the Centres for Disease Control and Prevention (CDC) climate and health framework (29) into a diagram. This comprehensive model (Figure 1) shows the complex pathways linking climate-related health impacts. The figure connects climate change events and health. For example, rising temperatures, extreme weather, rising sea levels, and increasing CO2 levels will affect water and food supplies. This, in turn, leads to malnutrition and diarrhoeal disease. The central areas of the diagram influence various health outcomes, including increased respiratory and cardiovascular disease from poor air quality and heat stress. Injuries and premature deaths are also included. The framework highlights the importance of preparedness and targeted public health interventions to mitigate these risks, serving as a foundation for identifying research priorities in local contexts. While interconnected, we distinguish climate change (global drivers), environmental degradation (ecosystem changes), and environmental health impacts (human outcomes such as air pollution exposure).

### Prioritization and analysis process

Using their local knowledge, participants engaged in a structured ranking exercise to express their relative priorities among the research topics derived from the CDC framework. Each participant was given 6 points to distribute across at least 3 topics, with a maximum of 3 points per topic. Points were represented by colored stickers: green (3 points), yellow (2 points), and red (1 point). This method encouraged participants to make trade-offs, revealing the depth of their preferences and highlighting the most urgent areas for research. This part of our work drew on participatory priority-setting approaches, such as the James Lind Alliance model, providing structured mechanisms for collaboratively identifying and ranking research uncertainties among stakeholders (30).

In Indonesia, 23 women attended the workshop; however, only 21 completed the prioritization exercise. In Malaysia, 17 women attended the workshops; however, only 14 completed the prioritization exercise and were included in the analysis. The overall ranked data value was calculated and mapped to the CDC framework in real time. To visualize the data, we presented the results in bar charts or tables and projected them onto the workshop room wall. To provide context for their priorities, a table discussion followed, during which the women explained the reasons behind their choices. This integration of qualitative data offers insights for interpreting and contextualizing the quantitative ranking results, providing a richer understanding of why certain topics were prioritized over others. We started by sharing Indonesian participants’ priorities and the points allocated to each topic, along with the reasons, followed by Malaysian women’s answers in the same format (priorities, points, and reasons).

We also added insights into each participant’s priority, based on cumulative evidence from three preceding discussion sessions, in which participants elaborated on daily life experiences, structural barriers, and context-specific health impacts. The insights were drawn from qualitative data analysis through thematic hand-coding of workshop transcripts, following the thematic analysis framework established by Braun and Clarke (31).

Coding was conducted at the country level, with each country dataset assigned two coders. To capture cultural and local nuances, at least one coder per country was a local researcher with direct familiarity with the sociocultural and linguistic context. To ensure analytical consistency, coders independently coded the same transcripts before engaging in structured cross-checking of respective analyses.

## Findings

### Indonesia

Among the 21 Indonesian women who voted, by marital status: 12 were married, 4 were single or childfree, and the remaining did not specify their marital status. By parental and caregiving status, 12 were mothers (with children from toddlers to teenagers), and some also cared for older parents. By living arrangement, 13 lived with immediate or extended family; others maintained separate households or lived independently due to work or caregiving. By location, most lived in urban or suburban areas (such as Jakarta and Bogor), while three were based in or connected to semi-urban regions (such as Sukabumi, West Java). By health status, seven reported physical or mental health issues. By occupation, six worked in health or research, six in grassroots or community sectors, and the remaining in education, trade, digital media, or climate governance.

**Figure 2:**
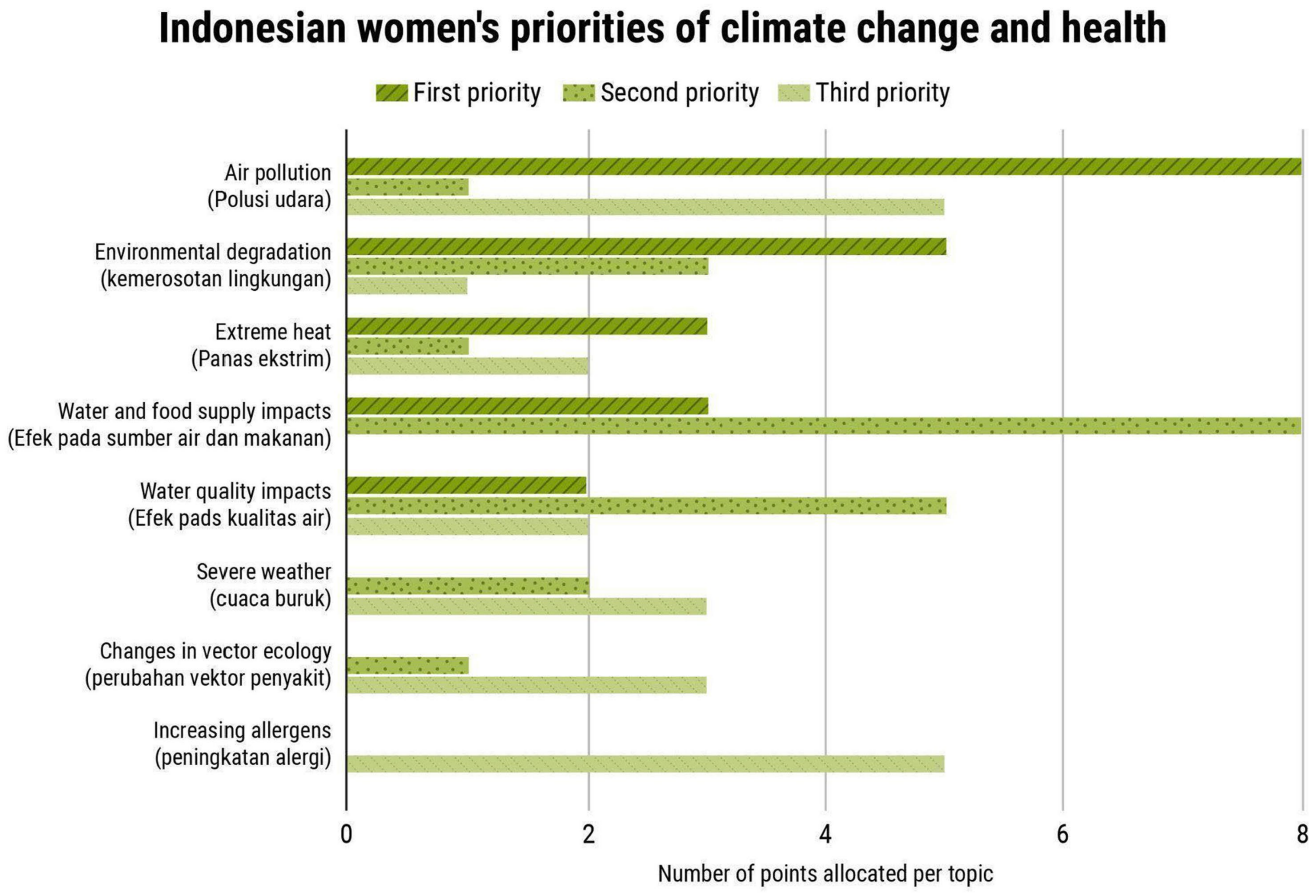
First-, second-, and third-ranked climate change and health research priorities identified by Indonesian women (*n* = 21). Figure: Impacts of Climate Change on Human Health. Source: Centers for Disease Control and Prevention (CDC), U.S. Department of Health and Human Services. This image is a work of the United States Federal Government and resides in the public domain. Image unmodified from the original.

**Figure 3:**
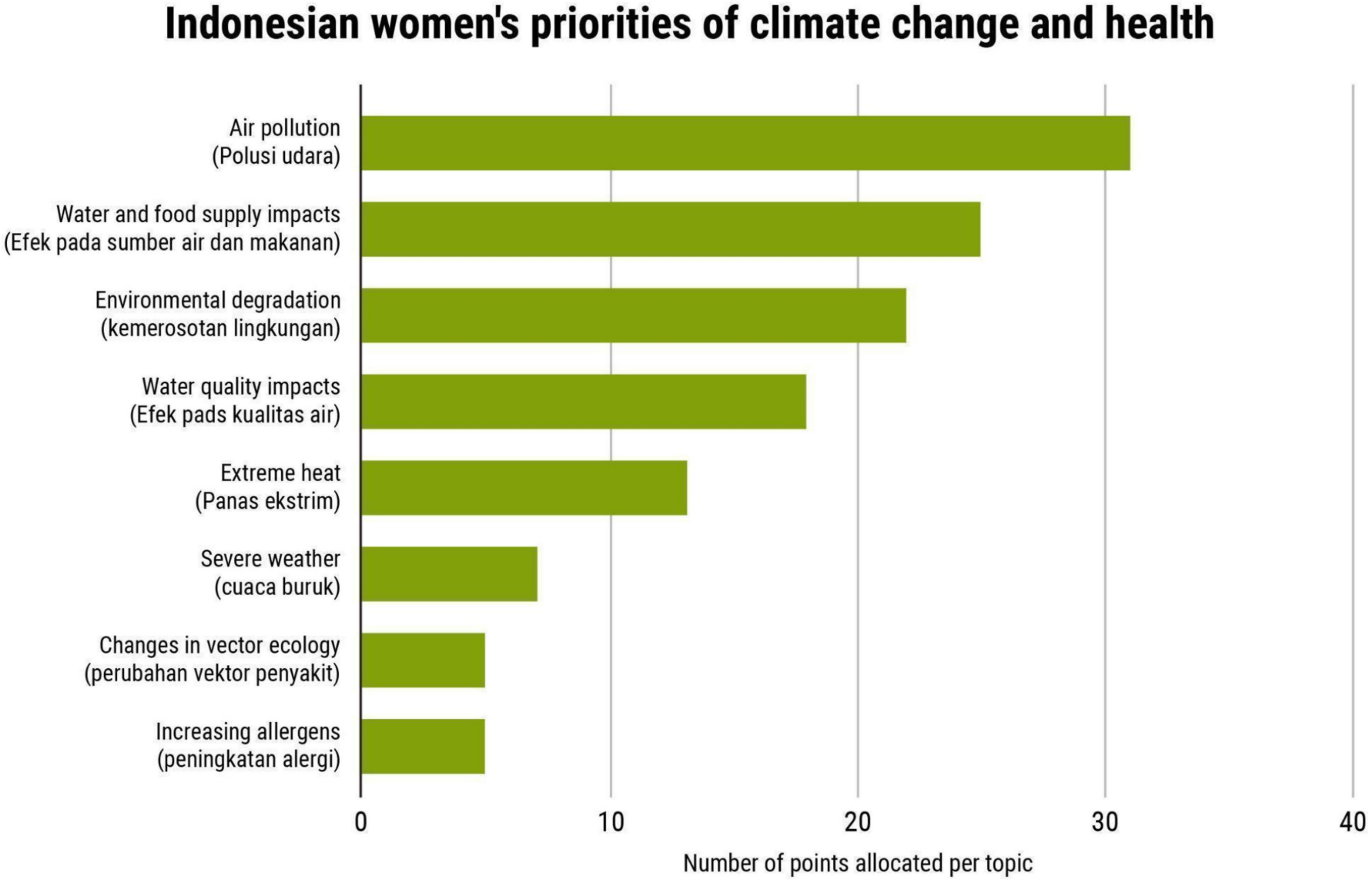
Total points assigned to climate change and health research priorities by Indonesian women during the prioritization exercise. Figure: Impacts of Climate Change on Human Health. Source: Centers for Disease Control and Prevention (CDC), U.S. Department of Health and Human Services. This image is a work of the United States Federal Government and resides in the public domain. Image unmodified from the original.

We will now review the results from the priority ranking exercise for each country. Figure 2 summarizes the 21 Indonesian women’s 1st-, 2nd-, and 3rd-priority research topics on climate change and health, while Figure 3 shows the tallied points across these priorities. Next, a summary of quotes provides context for the women’s concerns and attitudes.

#### Air pollution

While not all air pollution is related to climate change, it has become a top priority and received the highest overall score (31 points). Many women expressed worries about rising respiratory illnesses, especially among children and the older people, and the difficulty of avoiding both outdoor and indoor pollutants in dense urban areas. This reflects broader health issues in Indonesia, where poor air quality in cities like Jakarta and Surabaya greatly contributes to premature deaths and chronic diseases. The emphasis on air pollution by women highlights both immediate daily struggles and long-term health fears. It also underscores the urgent need for policies that cut emissions from transportation, industry, and forest fires while enhancing air quality monitoring and public health communication. Concerns about air pollution are closely tied to their roles as women and breadwinners, which require daily outdoor activities. For residents of Jakarta and nearby areas, and for people spending a majority of their time in outdoor spaces, either for work or using public transport, air pollution has been a persistent, troubling, and unresolved issue that contributes to ongoing respiratory issues such as chronic coughs.

Interestingly, one woman (IP4), a motorbike taxi driver who works daily on the streets and has experienced persistent coughing, which she attributes to air pollution, initially prioritized air pollution but chose water and food first, as she considers these her basic needs.

*Air pollution affects my health, causing coughing, because I work outside every day and have no government health insurance.*

*(IP4)*

One participant was a mother of two who works in government as a policymaker focusing on air pollution and drought-related impacts. She discussed how air pollution in Jakarta has led to a decline in bird populations, prompting people to keep as many as 10 ornamental birds in cages at home. These birds shed dust, dander, and droppings, but they cannot be kept outside due to the risk of theft. She said:

… *it's happening constantly, even in Jakarta. It's also hard to fix them; there's both indoor and outdoor pollution. In healthcare, we focus on indoor air... Often in dense neighborhoods, especially in Jakarta, they keep birds, which contributes to indoor air pollution issues [too]. (IP21)*

These accounts illustrate how air pollution is not only an environmental exposure but also a gendered burden, as women’s caregiving, informal work, and family health management roles increase both their exposure and their responsibility for managing its consequences. Women’s limited access to healthcare, occupational protections, and social support systems compounds their vulnerability to climate-related health risks.

#### Water and food security

Closely following air pollution were concerns about impacts on water and food supplies (25 points). Participants highlighted increasingly erratic rainfall, prolonged droughts, and contamination of water sources as drivers of food insecurity and malnutrition, which impose an economic burden and create a form of neo-colonization, forcing the country to depend on foreign aid. These challenges particularly affect women, who often bear primary responsibility for securing food and water for their families. The focus on this issue calls for strengthened climate-resilient agriculture, protection of local water sources, and community-based early warning systems for drought and flood events.

Participant IP9, who works in traditional markets and places high importance on food and water security, has a lifestyle closely connected to food systems vulnerable to climate variability. As a married woman in her late thirties, she describes herself as someone dedicated to ensuring that rural women’s experiences inform research and policy. She said:

*Water quality… declines, becoming dirtier and blacker, which affects food quality, such as rice… (IP 9)*

One participant, IP20, is an unmarried woman who faces extreme heat daily and lives alone with 18 cats, which often fall ill due to the heat. Described these dynamics as creating a form of “neo-colonization,” reflecting concerns about increasing dependence on external resources and reduced national self-sufficiency

*My second priority is water and food, which are global issues, including in Indonesia. Even rice is being imported now. Food security is crucial because without it, we could be colonized again. (IP20)*

IP4 operates as a motorbike taxi service. She lives with mental health issues and has a persistent cough. Despite her daily exposure to air pollution and its impact on her reproductive health she prioritized food and water over reproductive health. She said:

*Food and water are the main priority, then health, because it is difficult to find money, and many people are even in debt just to meet basic dietary needs.*

(IP4)

These findings demonstrate the need to improve women’s access to economic resources, land, and decision-making power, while strengthening supports that help them adapt to climate-driven disruptions.

#### Environmental degradation

Environmental degradation (22 points) ranked third among key concerns. Women associated issues such as deforestation, waste mismanagement, and loss of green spaces with rising temperatures, reduced air and water quality, and psychological stress. This comprehensive framing of environmental degradation as a health issue is significant; it highlights women’s awareness of the interconnectedness between ecological and human well-being, especially as it relates to their own bodies.

Most participants also agreed that environmental degradation is the root cause of various health impacts linked to climate change. Environmental damage, primarily driven by large industries, is a shared experience among women across Indonesia, affecting diverse regions of the country. This degradation affects not only urban women but also those living in coastal and forested areas.

An Indigenous woman, who studied planetary health and focuses on connecting the environment to health, said:

*Environmental damage is the biggest problem in Indonesia...For us Indigenous people, if nature is damaged, we lose sources of food, life, medicine, and so on.*

*(IP22)*

Participant IP3, a Gen Z social media campaigner and florist, shared a story about how mining for rare minerals, such as nickel for rechargeable batteries linked to climate solutions, has contaminated water sources, leading to the death of a woman.

…*in Halmahera, access to clean water is very limited due to nickel mining. [After a death] In one case, a woman’s body was found to contain nickel levels above the safe threshold.*

*(IP3)*

One participant, IP13, born into an upper-middle-class family and experiencing allergies and mental health issues, discussed how economic pressures on households often lead to higher rates of early child marriages and domestic violence.

*Behavioral changes due to climate change [cause an] increase in anger issues [which lead to] sexual and gender-based violence*

*(IP13)*

These impacts highlight the importance of ensuring women’s land rights, including them in environmental governance, and valuing Indigenous knowledge in decision-making processes.

#### Water quality and 5. Extreme heat

Participants observed increased incidences of waterborne diseases during floods, along with health burdens linked to limited access to safe drinking water. Participant IP1, a mother of two who manages household bills, a role uncommon for women in Indonesia shared her insights about water quality.

*Many small islands struggle with access to clean water. Some have even resorted to harvesting dew. Another issue is waste being dumped into the sea, such as septic tank waste. Even in Jakarta, the water is contaminated with E. coli, but most people aren’t aware of it.*

*(IP1)*

Some respondents also noted that water quality could vary across different regions of Indonesia. They recognized that water quality might not be a significant concern for most respondents living in urban areas.

*Water quality isn’t a top issue here (Jakarta), perhaps because it isn’t seen as a major problem in this area. But in places like Central Java and North Maluku, where there are nickel smelters, water issues are much more critical.* *(IP3)*

Meanwhile, extreme heat was recognized as an emerging yet increasingly familiar threat, especially for children, older adults, and household pets. Women caring for vulnerable family members noted that pregnant women, those working outdoors, and people living in informal housing without sufficient cooling would be most affected. Participant IP19, a mother of two and an active member of an NGO supporting urban poor communities, mentioned that rising heat was affecting her children’s health.

*Compared to 20 years ago, it’s much hotter now. The heat feels like it pierces your head. I only dared to take my child out after she turned 3. She once had a nosebleed from the heat. …My second child gets dizzy when going outside. My first child even had lung spots. So when they say it’s too hot to go out, we just stay in.*

(IP19).

Similarly, participant IP20, said:

*It can reach 38°C. Even my cats are affected. I had to take them to the clinic this week because they got mouth ulcers from sun exposure.*

*(IP20).*

A single woman who cares for ageing parents and desires a safer climate for her son said:

*unpredictable weather.. extreme weather, there's [so much]... uncertainty in life, … for people who want to work outside. How can people who want to go to school access it? It increases uncertainty. …The unpredictable weather, it's like a disaster in the end, ‘oh what if it's too hot there, what if it's flooded’, I think uncertainty just lowers the quality of life…*

*(IP15)*

Although ranked lower in overall priority, changes in vector ecology (5 points) and increasing allergens (5 points) remain recognized as areas of concern. Participants referenced rising dengue and malaria cases and increasing allergic reactions as potential signs of climate shifts.

As a mother of four sons, one participant acknowledges that the effects of climate change compound her duties. She said:

…*vector-borne diseases: children are exposed to dengue, malaria, etc., which leads to the mother taking care of the child when they get sick. (IP14)*

These experiences reveal how climate-related health risks such as extreme heat and poor water quality intersect with gendered caregiving roles, as women are often responsible for protecting children, older adults, and dependents from harm. Structural factors, such as inadequate housing, limited access to cooling infrastructure, and the unequal distribution of resources, further exacerbate these gendered vulnerabilities.

### Malaysia

**Figure 4:**
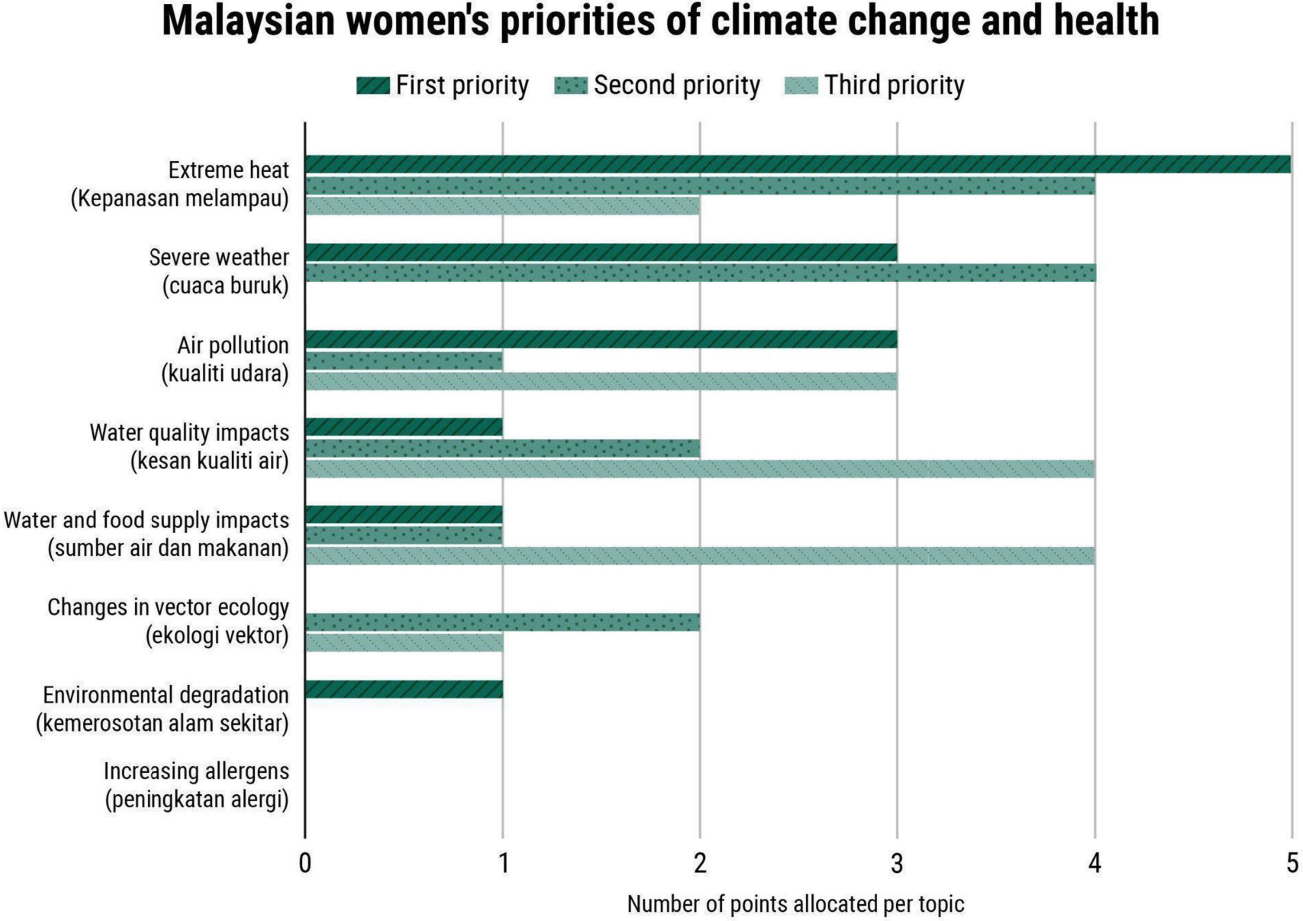
First-, second-, and third-ranked climate change and health research priorities identified by Malaysian women (*n* = 14). Figure: Impacts of Climate Change on Human Health. Source: Centers for Disease Control and Prevention (CDC), U.S. Department of Health and Human Services. This image is a work of the United States Federal Government and resides in the public domain. Image unmodified from the original.

**Figure 5:**
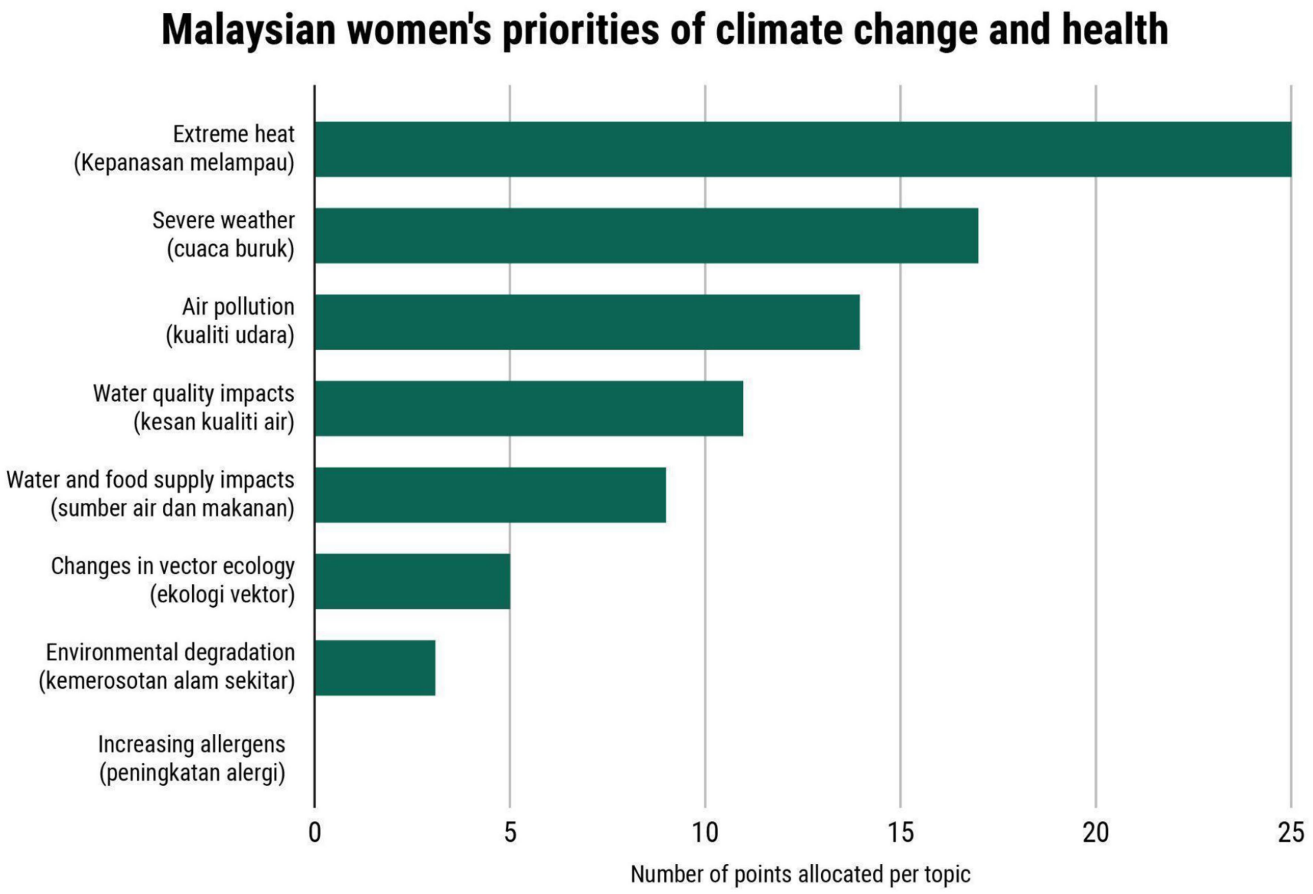
Total points assigned to climate change and health research priorities by Malaysian women during the prioritization exercise. Figure: Impacts of Climate Change on Human Health. Source: Centers for Disease Control and Prevention (CDC), U.S. Department of Health and Human Services. This image is a work of the United States Federal Government and resides in the public domain. Image unmodified from the original.

Of the 14 women in Malaysia who voted, five were married, and three of those women were mothers. Four still lived with their immediate (and extended) families, including parents, siblings, aunts, uncles, and grandparents. Four resided in rural areas, being from the *Orang Asli* and *Asal* communities, while 10 lived in urban areas. Two women were physically disabled, and three worked with migrant and refugee communities (Figures 4, 5).

#### Extreme heat

Extreme heat (25 points) was identified as the most pressing concern. Participants described how rising temperatures have become a daily struggle, especially for women working outdoors, pregnant women, older carers, and those living in homes without adequate cooling or ventilation. Common experiences included heat-related illness, dehydration, and fatigue. This concern primarily reflects the realities for urban women with limited adaptive infrastructure. To address these challenges, policies should focus on establishing accessible community cooling centers, developing guidelines for safe working hours during heatwaves, and investing in gender-sensitive urban planning. Specific actions include mandating heat-resilient housing designs, incentivizing property owners to enhance ventilation, and expanding green public spaces. Ensure public health messaging and adaptation programs address gender-specific heat risks.

Participant MP8 works for an NGO that supports communities in coping with climate disasters. She is married and has three children. In her role, she assists stateless and refugee women, she said:

*Women in perimenopause or menopause struggle to adapt to extreme heat, facing increased irritation, mental health challenges, and anxiety when children are exposed to hot weather.*

*(MP8).*

Regarding religious attire and heat, MP14 wears a headscarf, works for a faith-based NGO, and lives in a flood zone area:

*Muslim women wearing tudung face added difficulty coping with extreme heat.*

*(MP14).*

Extreme heat is experienced as a distinctly gendered burden, as women navigate its impacts alongside caregiving responsibilities, reproductive health concerns, and social expectations related to mobility and dress. These challenges are shaped by structural inequalities, including inadequate access to cooling infrastructure, financial constraints, and limited recognition of gender-specific health needs in climate adaptation planning.

#### Severe weather

Severe weather events, though sometimes overlapping with extreme heat, are categorized separately in the CDC diagram. In Malaysia, rainy and dry seasons shape these concerns. Severe weather, including floods, storms, and unpredictable rainfall, impacted crops, earning 17 points and ranking second. These events disrupt livelihoods, mobility, and mental health. Women, often acting as caregivers during crises, become more vulnerable during evacuations or in crowded shelters. MP11, who cares for a disabled sibling while managing her own health issues, shared concerns about floods, their effects on menstruating women, and the financial and emotional stress caused by severe weather.

*Flooding leads to anxiety, financial issues, and difficulties for menstruating women or those with children, especially among lower-income groups.*

*(MP11).*

Economic issues were also raised. MP1, who works for a sexual health NGO supporting women, expressed her concern about her fridge contents spoiling and the cleanup after floods. It is often women who clean up the home to make it livable again.

*When it thunders, the electricity in my house trips, risking food spoilage in the fridge. Sometimes I come home to a blackout and must restore power myself. I worry about the economic impact of ruined leftovers. Heavy rain can cause flash floods in the city, blocking roads. After floods, women clean the house, potentially exposing themselves to toxic pollution.*

*(MP1)*

A young *Orang Asal* woman (MP4) is a media student who worries about extreme weather drying out crops.

*Extreme drought and heavy rain destroy crops and cause hardship.*

*(MP4)*

MP4 stated that when the rain was too heavy after a drought, landslides occurred, which then polluted rivers, leading communities to act with the Government.

*Heavy rain after drought leads to landslides and contaminated rivers, prompting conflicts with the government over water supply.*

*(MP4).*

These accounts show how severe weather intensifies gendered inequalities, as women often take primary responsibility for caregiving, household recovery, and emotional labor during disasters. Structural factors such as insecure housing, limited finances, and a lack of gender-responsive disaster planning further worsen women’s climate risks.

#### Air pollution

Although not as highly ranked as in Indonesia, air pollution (14 points) remained a key concern. Women highlighted the link between poor air quality and rising rates of respiratory conditions among children and the older. Urban residents cited motor vehicle emissions and open burning as daily irritants that exacerbate existing health issues. A woman’s advocacy worker, who has experienced coastal erosion but now resides in Kuala Lumpur, said that when children fall ill, it is usually the mother who needs to take leave from work.

*Air pollution burdens women as they care for sick children.*

*(MP17).*

A married woman who uses public transport and has respiratory issues caused by poor air quality works in climate and ecological health. She is concerned that air pollution would most affect pregnant women.

*Air pollution is difficult for pregnant or chronically ill women and can affect their children.*

*(MP15).*

Furthermore, MP6, who works with B40 communities, said that we know very little about how B40 vulnerable groups cope with worsening air quality.

*Despite awareness, there is limited focus on how B40 women cope with air pollution.*

*(MP6).*

MP13, who advocates for women laborers, stated that factory workers might end up wearing masks for an extended period, which could cause breathing and heat-related issues.

*Air pollution requires mask-wearing, which, for some, such as factory and plantation workers, can lead to breathing difficulties and heat stroke. Some plantations provide only one long-term mask per worker.*

*(MP13)*

Air pollution is a gendered issue due to its intersection with caregiving and workplace exposures, particularly for women supporting children, older adults, and vulnerable community members. These experiences highlight inequities in access to clean environments, workplace protections, and health services for low-income and marginalized groups.

#### Water and food supply

Water quality impacts (11 points) and issues related to water and food supply (9 points) were considered mid-tier concerns. Participants shared experiences of water scarcity, contamination, and rising food prices linked to climate variability. Women’s traditional roles in household food preparation and water collection increase their exposure to these issues. These priorities highlight the need to improve infrastructure to ensure clean water access, including upgrading rural and urban water systems and enforcing stricter pollution controls. Support should be provided for climate-resilient agricultural practices, for instance, by offering technical training to women farmers and subsidies for sustainable inputs. Public communication strategies should be strengthened to provide timely guidance on food safety during climate events. There was a clear difference between the *Orang Asli/Orang Asal* participants and others, with water being a key priority. This is because Indigenous people live near rivers, which become polluted after extreme weather events worsened by climate change. Climate change impacts water quality in MP12’s area.

*Rivers are a direct resource. Logging has polluted them, turning the water the color of milky tea. While my community protested and logging was briefly halted, the river remains stagnant. We found a clean but small river and built a small dam with [NGO] funding. Some still use the polluted river’s muddy water, causing skin diseases. Now, with the new dam, my community finally has clean water, essential for daily tasks.*

*(MP12)*

MP3 is a married *Orang Asli* woman with three children. She works at an animal conservation center, feeding the animals with foraged forest food and making play structures and furniture for them. As a mother, she possesses traditional medicinal knowledge, foraging the forest for guava roots whenever her children are ill. She mentioned that water is linked to food scarcity, and when food poverty becomes an issue, it leads to conflicts within the household.

*Bad weather creates stress and household conflict over food scarcity.*

*(MP3).*

Water and food insecurity are closely tied to gendered roles within households and communities, with women disproportionately responsible for ensuring access to safe water and adequate food. These challenges are shaped by structural inequalities, including rural–urban disparities in infrastructure, marginalization of Indigenous communities, and limited inclusion of women’s perspectives in resource governance.

### Under prioritized yet relevant issues

Issues such as changes in vector ecology (5 points) and environmental degradation (3 points) received fewer votes, and increasing allergens (0 points) was not prioritized at all. During the discussions, women highlighted that rising allergen levels were linked to air pollution, for example, asthma. This again illustrates the perceived interconnectedness of climate and health. It also reveals an opportunity for public health and climate communication campaigns to fill knowledge gaps, particularly regarding emerging climate-health risks such as dengue outbreaks and biodiversity loss.

For one woman working for an NGO supporting women after a climate-related disaster, her NGO distributes food, water, sexual and reproductive health kits, and sanitary products. MP9 said they ranked the increase in allergens and vector ecology lower due to a lack of localized data.

*There were some priorities – like “increasing allergens” and “vector ecology changes” – that I hesitated to rank highly due to a lack of localized data or community awareness, rather than because of their importance. This highlights the need for further awareness and research on emerging or less visible health issues.*

*(MP9)*

MP10 lives with a disability and advocates for women with disabilities. She was the only participant who believed environmental degradation should take top priority because she noted that she had not seen any research on people with disabilities being displaced by environmental issues. This finding further emphasizes the need for individual and community-level planning in the research design stage.

*During disasters, people are forced to move, but there is no research on people with disabilities experiencing forced migration.*

*(MP10)*

On the topic of climate change and mental health, a zero-waste campaigner (MP5) who works with marginalized groups recollected an encounter with someone suffering from climate anxiety.

*[The] mental health aspect is not really looked at by our government…. I met a lady who was in a coma from an accident. When she recovered, she started to have climate anxiety, but she is not getting much health support. Many do not know how climate contributes to mental illness, and she lives with that anxiety.*

*(MP5)*

MP9 has worked with marginalized communities affected by climate-related disasters. She works closely with refugees and ranks forced migration as her top priority:

*Based on the chart [CDC diagram], we see that there’s very little vote on forced migration. We talked about different ways of living in cities and different [intersectional] groups. Nobody acknowledges forced migration in Malaysia. … Especially on mental health, it has a burden not just adapting to the country itself, but also the changing climate and weather. We work a lot with refugee populations and clinics dedicated to them, and they pose different health and mental health issues. It’s going to be a lot more to bear for these populations.*

*(MP9)*

## Discussion

Interpreting these findings through an intersectional and feminist climate justice lens reveals how women’s experiences are shaped not only by environmental exposures but also by structural inequalities, including gendered division of labor, unequal access to resources, and exclusion from decision-making spaces. These findings align with ecofeminist arguments (32) that environmental degradation and gender inequities are mutually reinforcing, particularly in low- and middle-income contexts. Our findings align with global frameworks for gender-responsive climate action, such as the UNFCCC (12) Gender Action Plan, which emphasizes the need to integrate gender considerations into climate adaptation, health systems, and decision-making processes. Our study extends this framework by demonstrating how women’s lived experiences in Indonesia and Malaysia can inform more context-specific, locally grounded approaches to gender-responsive climate–health policy.

Our study was conducted in Southeast Asia (Indonesia and Malaysia), where an intersectional, life-course approach was adopted to examine how multiple identities and intersecting demographic factors, such as age, socioeconomic status, rurality, and caregiving roles, interact to shape women’s exposure to and resilience to climate and environmental risks. This study has identified several critical climate issues facing women, including air quality and extreme heat. The study has also shown that women are holders of knowledge and potential solutions to climate and health research inequalities, as well as sources of wisdom to support climate adaptation. A key contribution of this study is the inclusion of perspectives from marginalized groups, including Indigenous women, women with disabilities, and those working with refugee and low-income communities. These perspectives provide critical insight into how climate-health risks are experienced unevenly, revealing gaps in existing policies that often fail to account for intersectional vulnerabilities. In their different ways, they demonstrate agency and resilience in the face of almost existential crises. Policymakers and researchers should consider how these holders of knowledge can inform not only a nuanced understanding of environmental degradation and climate risk but also the most likely future mitigation strategies with the greatest positive impact.

These findings match studies from other Global South contexts, where women’s climate vulnerabilities stem from caregiving roles, informal employment, and limited access to adaptive resources (23). Our findings further clarify that, in Indonesia, air pollution is the primary concern, while in Malaysia, extreme heat takes precedence. This difference reflects local environmental and socio-economic conditions.

Methodologically, we have several points and potential limitations to raise. We tried to mitigate this, but social desirability bias may still be present. We noted that before the ranking process began, each table discussed its preferred priorities, what they planned to select, and why. After the ranking was conducted, a further table discussion followed as we projected the live votes being counted. At this point, a woman in Malaysia said she wanted to change their priorities. On this occasion, the wrong color (value sticker) had been placed, but the voting results remained the same. This made us reflect that it is possible that potential influence of facilitation and group hierarchies on prioritization could have occurred, although we raised at the start of the ranking that everyone is different and will prioritize the issues differently according to their life and situations. We also note that some women who had paid jobs as community workers may have ranked priorities based on their advocacy positions rather than their home and personal situation (even though we stated that they could use both their home and professional lives to conduct their choice of ranking). Finally we also wish to acknowledge that in our quest to simplify the process, forced ranking does simplify very complex issues and limiting the number of points may have constrained their expression.

In the future, in a similar setting, researchers could explore independent and private voting to minimize any influence or bias. However, given this was principally a qualitative study with the voting’s purpose largely to prompt discussion, we do not see any impact on our findings. Future researchers may wish to use the Delphi method to explore the extent to which women might change their votes, as we did not ask for feedback on the voting results after we had reviewed and discussed the overall results. The Delphi Method is a structured forecasting technique that gathers expert opinions anonymously through multiple rounds of questionnaires to reach a consensus on complex issues. It is especially valuable for decision-making in uncertain environments, as it allows diverse perspectives to converge without the influence of dominant voices (33). This study has several limitations. First, we used purposive sampling in Indonesia and Malaysia; despite reaching out to a broad range of NGOs to share our research opportunity, some women may not have had the chance to participate. Second, the workshop-based approach may be influenced by social desirability and group dynamics, which could shape participant responses and limit the expression of more sensitive experiences. Third, differences in language, culture, and context across the two countries may affect the comparability and interpretation of the findings, despite efforts to ensure consistency in facilitation and analysis. Fourth, while the study focuses on women’s experiences, it does not fully account for intersectional differences within this group, including those related to socioeconomic status, ethnicity, and geographic location. Finally, the data reflect perceptions at a single point in time and may not capture the changing nature of climate-related health risks and adaptive responses.

The strength of our research is that we have engaged active knowledge holders whose lived expertise remains an underutilized asset in climate adaptation governance across Southeast Asia. However, translating their expertise into formal decision-making requires more systemic efforts and long-term investment. For instance, in Indonesia, *Musrenbang* (Development Planning Conference) a legally mandated multi-stakeholder development planning forum that starts at the village level remains a persistently underutilized entry point for women’s climate health advocacy. Specifically, women’s participation in these forums is only around 10–20 per cent, and their voices are rarely included in the decisions. This exclusion is mirrored at higher levels, including the legislature. Despite Indonesia passing a law in 2008 establishing a 30 per cent quota for women in political activities, women remain excluded from decision-making processes due to deeply held patriarchal values. Addressing this challenge requires change, not just in establishing forums that tokenistically invite women to attend, but also in how women’s experiences and ideas are incorporated into climate change solutions within these forums.

This is where academics and civil society organizations play a crucial role. In particular, community-based participatory research (CBPR) functions as a partnership that can build capacity and drive policy change by equitably engaging diverse partners. Through this approach, participants’ knowledge, self-efficacy, and advocacy skills are enhanced. Furthermore, investing in participatory research is necessary, as it increases women’s capacity to design climate-health literacy tools and helps community members translate their climate change experiences. Ultimately, this process empowers advocacy.

Further, while the CDC model offered an important conceptual framework for understanding climate-related health risks, upon reflection, we realized that it did not fully capture the nuanced and specific vulnerabilities experienced by our participant women. Although disability, forced migration, and mental health were not highly prioritized in the ranking exercise, women’s narratives revealed these issues as intersecting and amplifying vulnerabilities. Climate-related displacement was described as particularly exclusionary for people with disabilities, given persistent barriers to safe evacuation, accessible shelter, and continuity of care. Mental health impacts emerged as both a consequence of climate stressors and displacement, especially for women with caregiving responsibilities. The under-prioritization of these themes reflects gaps in recognition and data rather than limited lived significance, emphasizing the need for intersectional, life-course-responsive climate–health adaptation. This was particularly evident when we reviewed the model from a life-course perspective, considering climate–health experiences in relation to intersecting demographic characteristics such as age, reproductive stage, pregnancy, older age, and women’s subsequent caregiving roles and responsibilities, as well as their access to health services. Climate change disproportionately affects vulnerable populations, and women, especially in LMICs, experience layered and intersecting risks that are often insufficiently addressed. For example, evidence indicates that pregnant women are physiologically less able to regulate body heat, making extreme heat exposure dangerous for both mother and baby and linked to pregnancy complications (34). Additionally, the CDC diagram does not distinguish between different types of vulnerabilities, particularly those experienced by women throughout their life cycle. Viewing women’s vulnerabilities through the lens of the female life-course cycle reveals that they encounter distinct risks from puberty, adolescence, reproductive years, to post-reproductive and older age stages. Therefore, adopting a life-course approach to public health adaptation is crucial for ensuring equitable access to health services and treatments. Moreover, girls and women living in climate-affected environments with limited access to clean water often struggle with adequate hygiene and sanitation, impacting menstrual health, family planning, and access to contraceptives (35). Cultural and religious norms further heighten women’s vulnerability. Dress codes, religious attire, mobility restrictions, requirements for spousal consent, and gendered household roles can severely limit women’s ability to make autonomous decisions or access emergency services and evacuation centers during disasters such as floods or heatwaves (36).

Finally, demographic and socio-economic disparities such as income level, education, geographic location (e.g., rural versus urban, archipelagos versus continental), and the burden faced by female-headed households significantly influence women’s climate resilience. Studies from Southeast Asia have shown that women living in rural and agricultural households, especially those with lower income and education levels, bear a disproportionate burden from extreme weather events such as floods and heatwaves. These events are sometimes regarded as less of a climate-change priority (because they occur constantly). In the long term, they often lead to increased food insecurity, malnutrition, reduced access to essential sexual and reproductive health services (e.g., contraceptives and antenatal care), and higher rates of gender-based violence during and after climate disasters. These concerns are supported by emerging evidence linking environmental degradation, global supply dependencies, and health inequities in low- and middle-income countries, particularly where climate-related disruptions affect food systems and reproductive health outcomes (35, 37). It has also caused climate anxiety for women who bear the primary role as family caregivers (38). While the CDC model served as a foundational tool, we argue that it requires significant adaptation to support gender-sensitive, equity-focused public health responses.

## Conclusion

Table 2 highlights both shared and context-specific climate–health research priorities among women in Indonesia and Malaysia. Indonesian women prioritized air pollution and resource security. In contrast, Malaysian women identify extreme heat and severe weather as key threats.

**Table 2:** Summary of top climate change and health research priorities identified by women in Indonesia and Malaysia.

**Rank**	Indonesia (*n* = 21 women; prioritisation exercise)*	Malaysia (*n* = 14 women; prioritisation exercise)*
1	Air pollution (respiratory illness, indoor and outdoor exposure, impacts on children and older adults)	Extreme heat (heat stress, dehydration, impacts on work, caregiving, pregnancy, and menopause)
2	Water and food security (erratic rainfall, drought, food affordability, water contamination)	Severe weather events (flooding, storms, drought, livelihood disruption, mental health impacts)
3	Environmental degradation (deforestation, waste, industrial pollution, loss of ecosystems affecting health and wellbeing)	Air pollution (respiratory illness, caregiving burden, occupational exposure)
4	Water quality (access to safe drinking water, flooding-related contamination)	Water quality (river pollution, access to clean water, especially in Indigenous communities)
5	Extreme heat (heat stress affecting children, older adults, outdoor workers, informal housing)	Water and food supply (food prices, food security, household impacts)
Lower-ranked but noted	Vector-borne diseases; increasing allergens	Vector-borne diseases; environmental degradation; increasing allergens (not prioritized)

*Qualitative discussions involved all participants; prioritization was completed during the workshop exercise as described in the Methods.

These findings underscore the importance of gender-responsive climate–health research and policy that centers lived experiences. Women’s perspectives provide critical insight into locally relevant priorities and potential pathways for more equitable and effective interventions.

### Recommendations

#### Incorporate women’s voices and priorities into climate health policy

**Figure 6:**
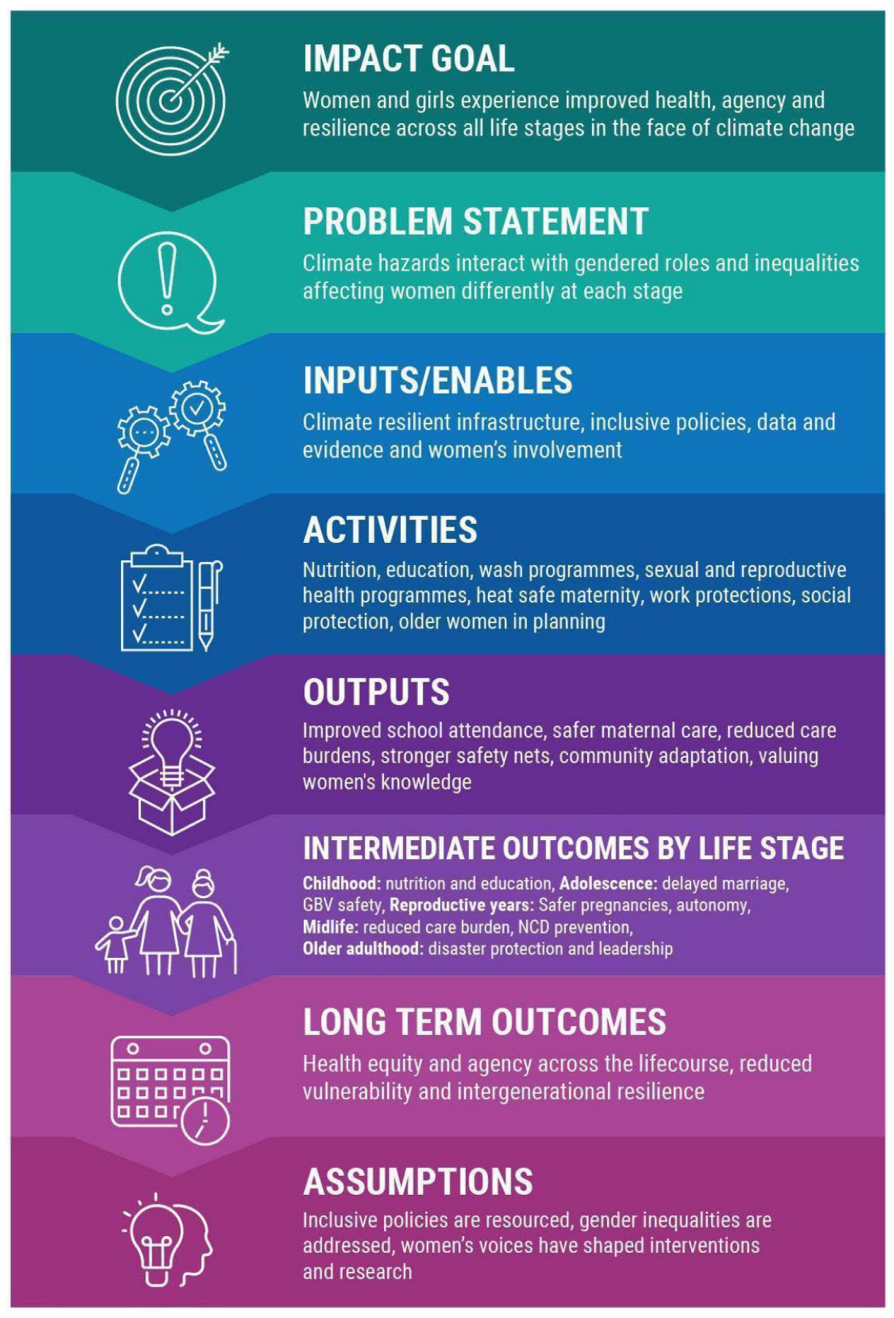
Conceptual framework illustrating climate change impacts across the female life-course in low- and middle-income country (LMIC) settings. Figure: Impacts of Climate Change on Human Health. Source: Centers for Disease Control and Prevention (CDC), U.S. Department of Health and Human Services. This image is a work of the United States Federal Government and resides in the public domain. Image unmodified from the original.

Ensure that national and subnational strategies for climate adaptation and public health explicitly address women’s most urgent concerns, including heat stress, air pollution, extreme weather, and resource insecurity. Women also perceive climate change impacts differently and are more likely to concentrate on issues that affect not only themselves but also their communities. The women in Indonesia and Malaysia who participated in our research included local and cultural concepts that were previously unexamined in global North research. These should be integrated into existing frameworks such as Nationally Determined Contributions (NDCs), health emergency response plans, and local climate action roadmaps, as shown in Figure 6: climate change across the female life-course – a research framework.

#### Invest in gender-responsive solutions

Allocate resources to implement community-based programs that reflect women’s lived experiences. This includes public cooling infrastructure and heat-health mitigation campaigns that women can easily access in languages they understand. To minimize the health impacts of air pollution, clean-air enforcement policies are urgently needed, along with greener urban spaces. Safe drinking water and climate-resilient food systems need to be invested in so that women can continue to support their families and communities without compromising their health and that of their immediate families. Disaster relief initiatives need to consider intersectional gender issues, such as support for disabled or single mothers.

#### Amplify women’s leadership

Promote women’s leadership in climate governance and health systems at the grassroots and policy levels. Their knowledge must shape not only diagnostics but also the design, implementation, and monitoring of adaptation strategies.

#### Expand participatory and gender-sensitive research priorities

Support further research to deepen understanding of how climate risks affect women’s health, especially among rural, Indigenous, and low-income communities. Prioritize participatory methods that center and validate women’s knowledge and generate actionable data for policy making.

#### Create safe spaces for women

These spaces are not merely physical but also institutional and relational spaces where women can speak openly, share lived experiences, and be supported without judgment or hierarchy. This could be achieved by building a community of practice among women working in this space. This sense of togetherness is deeply rooted in Southeast Asian cultural values and continues to shape how communities work together to face challenges and care for one another.

#### Centre the voices of women as caregivers

Women will often prioritize others, their children, their husbands, their older parents or in-laws, the sick and the disabled living with them or around them. Therefore, asking women about who they are caring for will offer clearer insight into household dynamics and what society needs for a more just and equitable future for all.

#### Strengthen regional collaboration

Our findings point to the need for platforms for shared learning and action between Southeast Asian countries to elevate gender and health equity in climate policy. Regional institutions should facilitate cross-border dialogue and policy harmonization rooted in the priorities of frontline women. A just climate future demands action rooted in equity, care, and lived experience. Women’s voices are not marginal; they are vital. Listening to them is only the first step. Acting on their priorities is our shared responsibility.

## Data Availability

The raw data supporting the conclusions of this article will be made available by the authors, without undue reservation.
